# A feasibility trial of an intervention in alcohol dependence for structured preparation before detoxification versus usual care: the SPADe trial results

**DOI:** 10.1186/s40814-021-00880-6

**Published:** 2021-07-29

**Authors:** Christos Kouimtsidis, Ben Houghton, Heather Gage, Caitlin Notley, Vivienne Maskrey, Allan Clark, Richard Holland, Anne Lingford-Hughes, Bhaskar Punukollu, Morro Touray, Theodora Duka

**Affiliations:** 1 Surrey & Borders NHS Trust, Research and Development, Abraham Cowley Uni, Chertsey, Surrey, KT16 0AE UK; 2 University of Surrey, 388 Stag Hill, Guildford, GU2 7XH UK; 3 Norwich Medical School, University of East Anglia, Norwich Research Park, Norwich, NR4 7TJ UK; 4 University of East Anglia, Norwich Research Park, Norwich, NR4 7TJ UK; 5 University of Leicester, University Road, Leicester, LE1 7RH UK; 6 Imperial College London, Burlington Danes Building, Hammersmith Campus, 160 Du Cane Road, London, W12 0NN UK; 7 Camden and Islington NHS Foundation Trust, St Pancras Hospital, 4 St Pancras Way, Kings Cross, London, NW1 0PE UK; 8 University of Sussex, School of Psychology, Falmer, Brighton, BN1 9RH UK

**Keywords:** Alcohol dependence, Detoxification, Structured preparation, Pre-habilitation, SPADe

## Abstract

**Background:**

Individuals who are ‘moderately’ or ‘severely’ dependent consume alcohol at levels that are likely to have a severe impact on their own health and mortality, the health and behaviours of others (family members) and to have economic and social implications. Treatment guidelines suggest that treatment needs to be planned with medically assisted withdrawal (also referred to as detoxification), and aftercare support but outcomes are poor with low proportions engaging in after care and high relapse rates. An approach of structured preparation before alcohol detoxification (SPADe) puts an emphasis on introducing lifestyle changes, development of coping strategies for cravings, stress and emotions as well as introducing changes to the immediate family and social environment in advance of alcohol cessation. Such a pre-habilitation paradigm compliments the established treatment approach. The key research question was: can we design a large scale, randomised controlled trial (RCT) that will answer whether such an approach is more effective than usual care in helping individuals to maintain longer periods of alcohol abstinence?

**Methods:**

This is a pragmatic, parallel, two-arm, feasibility RCT comparing SPADe and usual care against usual care only in maintaining alcohol abstinence in adults with alcohol dependence receiving care in two community addiction services in London. Feasibility outcomes, exploration of primary and secondary clinical outcomes and health economic outcomes are analysed. The trial follows the guidelines of phase 2 of the Medical Research Council (MRC) for complex interventions.

**Results:**

We were able to recruit 48/50 participants during a period of 9 months. Retention in the trial for the whole period of the 12 months was 75%. Treatment compliance was overall 44%. Data completion for the primary outcome was 65%, 50% and 63% at 3, 6 and 12 months, respectively. The intervention group had more days abstinent in the previous 90 days at the 12 months (*n* = 54.5) versus control (*n* = 41.5).

**Conclusions:**

The results of this feasibility trial indicate that with the appropriate modifications, a full multicentred trial would be possible to test the effectiveness and cost-effectiveness of a pre-habilitation approach such as the SPADe group intervention in addition to usual care against usual care only.

**Trial registration:**

Name of registry: ISRCTN; Trial Registration Number: 14621127; Date of Registration: 22/02/2017.

## Key messages regarding feasibility

- What uncertainties regarding feasibility existed prior to the study? In 2005 in the UK, an innovative group intervention for preparation before alcohol detoxification, based on CBT Relapse Prevention interventions, developed by members of the research team, reduced dropouts during the detoxification process and improved outcomes at 1, 3 months and 6 months. Qualitative evidence found that ‘regaining control’ was the main learning point across all group sessions of the programme. However, these findings were from small naturalistic studies. Evidence was required on whether structured preparation before detoxification rather than detoxification alone improves short- and long-term treatment outcomes and therefore whether the whole treatment paradigm should shift.
- What are the key findings on feasibility from this study? The rate of recruitment was higher when the research assistants were on site, which was supported by the qualitative interviews of staff. Retention in the trial for the whole period of the 12 months was 75%, indicating strong acceptability in the study population. Treatment compliance was overall 44%, which is considered acceptable for a population attending addiction services. Data completion for the primary outcome was 65%, 50% and 63% at 3, 6 and 12 months, respectively, with more follow-ups taking place over the phone.
- What are the implications of the findings on the design of the main study? In a future RCT, we would randomise individuals at a later point once they are retained in the service for a brief period and so demonstrating some clear commitment to treatment. Furthermore, the intervention should be better standardised with the expectation that participants attend consecutive weekly sessions, within a maximum period of 12 weeks. Missed sessions would be offered by one of the facilitators on an individual basis. Most importantly there would be more enhanced communication and cooperation between key workers and group facilitators for a pro-active and more effective management of participants at risk of dropping out. Support of group facilitators should be allocated to local supervisors to increase hands-on support, to reduce contamination between the intervention and usual care.

## Background

Harmful use of alcohol continues to be a global health problem and tackling the impact of harmful and dependent drinking is a key global public health priority. Global estimates suggest that one in five adults report at least one occasion of heavy drinking in the past month [1]. Individuals who are ‘moderately’ or ‘severely’ dependent consume alcohol at levels that are likely to have a severe impact on their own health and mortality, the health and behaviours of others (family members) and to have economic and social implications [2]. In the UK, hospital admissions attributable to alcohol in 2017–2018 remained similar to the previous year (338,000). However, this is 15% higher than 10 years ago [3]. Alcohol misuse is linked directly to a range of health disorders. Cancer, accidental injuries and mental health problems remain the main alcohol directly related diagnosis leading to a hospital admission [3]. More than 5000 individuals died directly from alcohol use in 2016 in the UK, which is 6% higher than 2011 [3]. However, in the year 2018–2019, in the UK those in treatment for alcohol alone neither increased nor decreased on the previous year. This follows large year-on-year declines from a peak of 91,651 in 2013 to 2014 [4].

Treatment guidelines in the UK for moderate to severe alcohol dependence have largely remained the same for the past decade. They suggest that treatment needs to be planned, with up to four motivational sessions focusing on treatment engagement and development of aftercare support, followed by medically assisted withdrawal (also referred to as detoxification), and finally aftercare support, which should include pharmacological treatments, psychological treatments and access to self-help interventions and support [2]. Benzodiazepines are normally prescribed during detoxification to reduce the overt symptoms of alcohol withdrawal (sweating, tremor) as well as prevent potentially life-threatening complications (e.g. convulsions, delirium tremens) [2]. However, these drugs do not prevent alcohol craving, relapse back into alcohol consumption and other long-term effects on mental functioning [2, 5]. Outcomes from detoxification are often poor with low proportions engaging in after care [6] and are associated with high relapse rates [2]. There is accumulating evidence from animal and human studies that exposure to multiple detoxifications is associated with cognitive and behaviour changes in tasks indicating impairment in conflict resolution, i.e. what alcohol dependent individuals experience when there is a conflict between the intention to abstain from drinking and the desire to drink. In addition, those individuals show an increased sensitivity to stress and heightened craving. Together, these factors may contribute to relapse and might compromise the effectiveness of aftercare support [7–12].

In the light of the evidence indicating risks associated with detoxification, one potential approach to maximise effectiveness of each detoxification may be following a pre-habilitation treatment approach that compliments the established treatment and rehabilitation approach. Such an enhanced paradigm considers and plans for the management of the risks associated with detoxification as well as the risk factors associated with relapse. Such an approach involving structured preparation puts emphasis on introducing lifestyle changes, development of coping strategies for cravings, stress and emotions and introduces changes to the immediate family and social environment in advance of alcohol cessation. There is currently no guidance specifically on preparation for the detoxification process apart from general guidance on care coordination and case management [2]. Absence of specific guidance reflects the lack of developed interventions in this area. Evidence is required on whether structured preparation along the lines of pre-habilitation principles before detoxification rather than detoxification alone improves short- and long-term treatment outcomes and therefore whether the whole treatment paradigm should shift. Before embarking on a full trial of the effectiveness of such a structured preparation, there is a need to undertake a feasibility study to establish key parameters that influence trial design such as recruitment, adherence to the intervention, retention, and sensitivity of alternative outcome measures.

A literature search of PubMed Central using alcohol relapse prevention treatment-related MeSH terms undertaken in June 2014, found (i) that group interventions with diverse theoretical bases are considered to be more cost-effective than one-to-one interventions; and (ii) cognitive behaviour therapy (CBT) relapse prevention interventions are well supported by evidence [13]. In 2005 in the UK, an innovative group intervention for preparation before alcohol detoxification, based on CBT relapse prevention interventions, developed by members of the research team, reduced dropouts during the detoxification process [6, 14] and improved outcomes at 1, 3 months and 6 months [15]. However, these findings were from small naturalistic studies. Qualitative evidence found that ‘regaining control’ was the main learning point across all group sessions of the programme [16].

This feasibility study builds on the above preliminary evidence by refining the preparatory intervention and assessing the feasibility of conducting a large-scale evaluation (Structured Preparation before Alcohol Detoxification: SPADe) for individuals with moderate to severe alcohol dependence, as an adjunct to usual care, consisting of planned detoxification and aftercare.

The intervention under investigation in this study is based on Plans, Responses, Impulses, Motives, Evaluations (PRIME) theory of motivation [17] and Social Learning theory [18]. PRIME theory is a synthetic theory of motivation from conscious decision-making through to classical and instrumental learning processes. The theory understands the motivation system as a system of forces and operates at five levels of complexity. Any of these can function abnormally in addiction. As a person moves from reflex responses, through impulses, then motives and evaluations, greater flexibility of responding, consideration of a wider range of factors and anticipation of future consequences are allowed. At the highest level, plans allow action sequences to be prepared in advance of the circumstances when they are needed. This is consistent with the overall pre-habilitation approach that aims to prepare people to cope with life after the removal of alcohol. Social learning theory introduces the concept of self-efficacy that underlies the use of psychological interventions aiming to reverse the development of automatised behaviour and associated loss of control, such as CBT relapse prevention interventions. The intervention combines the long established (in alcohol treatment) ethos of group intervention, and follows the biological principle of homeostasis, which is disturbed with prolonged alcohol use [19], to help individuals regain control over their drinking as the first step towards lifelong sustainable abstinence.

## Methods

### Aim

The key research question was: can we design a large scale, randomised controlled trial (RCT) that will answer whether the SPADe intervention is more effective than usual care in helping adults to maintain longer periods of alcohol abstinence? The feasibility trial compared the use of SPADe with usual care against usual care alone in two sites and enabled us to do the following:

1. Measure the number of eligible participants, willingness of clinicians to recruit participants, recruitment rate, loss to follow-up, adherence to the intervention and standard deviation of the primary outcome measures. This will ultimately inform the sample size calculation for a multicentre clinical trial.

2. Determine the acceptability of randomisation to service users, through its effect on recruitment, dropout rates and via qualitative interviews.

3. Determine the appropriateness and the acceptability of the outcome measures to participants to explore the suitability of our chosen primary and secondary outcome measures; percentage of days abstinent, service use and health-related quality of life.

4. Estimate the time needed to collect and analyse baseline and outcome data.

5. Explore the utility of the health-related quality of life instrument (EQ-5D-5L) (see outcome measures below) in allowing the estimation of quality adjusted life years in the sample, as well as exploring other aspects of cost-effectiveness assessment.

Reduction in subjective measures of alcohol dependence and craving as well as improvement in objective measures of mental functioning were also explored.

Finally, we have conducted qualitative interviews with participants and service providers, to assess the acceptability of the treatment and to explore their experience of the treatment including any barriers and/or facilitators to taking part in the study. Findings from these interviews will enable us to refine the SPADe intervention and the design of the future definitive RCT.

### Design

The study design is reported in detail elsewhere [20]. This is a parallel, two-arm, feasibility RCT comparing the clinical and cost-effectiveness of SPADe and usual care, against usual care only, in maintaining alcohol abstinence in adults with alcohol dependence receiving care in the community. The trial followed phase 2 Medical Research Council (MRC) guidelines for complex interventions [21] and includes adaptation of the intervention, feasibility study including health economics and process evaluation.

### Setting

Participants were recruited from specialist alcohol community services offering recovery orientated treatment for individuals with alcohol use disorders (AUD). Recruitment took place in two sites in London, both offering the intervention and usual care, to explore challenges associated with implementation of the intervention across two different treatment services. Both sites were run by a partnership between a third sector organisation and the National Health Service (NHS), a common funding model in the UK. The two sites had different integration levels; site 1 was fully integrated with a single management and clinical governance system; site 2 had two parallel systems. Notably, site 1 had been offering the intervention for several years as part of the standard treatment pathway, whereas in site 2 the intervention had to be added to the clinical pathway. This meant that in site 1 those randomised to control did not follow the established local treatment pathway but were provided with ‘usual care’ as provided elsewhere in London.

### Participants

#### Inclusion criteria

1. Presentation to either of the two alcohol services in London, Hounslow (site 1) and Camden (site 2) seeking abstinence from alcohol.
2. Alcohol dependence (moderate to severe), scoring 16 and above on Severity of Alcohol Dependence Questionnaire (SADQ) (see outcome measures below). This level of dependence indicates that it would be clinically appropriate to receive a medically assisted detoxification [2].
3. Stated intention to stay in the area within the time period of the intervention.
4. Willingness to be part of a group intervention if randomised to receive it.

#### Exclusion criteria

1. Age less than 18 (as not usually treated by specialist alcohol services).
2. Pregnancy: pregnant women need urgent intervention to withdraw from alcohol, due to the effect of alcohol on the foetus.
3. Known terminal illness with life expectancy of less than 6 months.
4. Severe medical condition that requires urgent medical admission, which would lead to an unplanned medically assisted withdrawal.
5. Severe cognitive impairment that compromises capacity and/or ability to participate in a group intervention.
6. Acute stage of a severe and enduring mental illness (schizophrenia, bipolar affective disorder, recurrent depressive disorder: current episode severe), when acute symptomatology compromises a service user’s ability to participate in a group intervention.

### Assessments

Participant assessments were conducted at baseline and at 3, 6 and 12 months by the research assistants who were trained in assessing capacity and obtaining consent, administering the questionnaires and interviewing participants. A window of 2 weeks either side of assessment time points was allocated to maximise engagement with follow up.

### Feasibility measures

Feasibility outcomes were (I) recruitment and retention rates: monthly monitoring of the number of alcohol dependent individuals accessing services during the recruitment period of the study; how many meet the eligibility criteria; and how many were invited and accepted into the study and retained in each group for the full 12 months. (II) Compliance with treatment: number of SPADe sessions attended (for the intervention arm) using the facilitator’s record of attendance. (III) Data collection and completeness: attendance for assessments and completeness of instruments; loss to follow up and missing data for all outcomes were analysed.

A variety of possible outcomes were used at each time point (3, 6 and 12 months post randomisation), so the primary outcome for the future trial could be identified and sample size calculations to be conducted, including duration of continuous abstinence with no incidents of lapse or relapse; percentage of days of abstinence (PDA) (both via self-report using Time Line Follow Back method) (TLFB) [22]; and time to relapse (from stopping alcohol to first day of alcohol use; also as defined by self-report).

### Secondary outcomes

Secondary outcomes were measured using validated instruments at 3-, 6- and 12-month post randomisation:

1. Severity of Alcohol Dependence Questionnaire (SADQ), a 20-item self-completion questionnaire, scores range 0 to 60, (16 to 29 indicates moderate severity, above 30 indicates severe dependence) [23].

2. Alcohol Urge Questionnaire (AUQ), an eight-item self-completion questionnaire containing three domains of drinking urges: desire for a drink; expectation of positive effect from drinking; inability to avoid drinking, with a score range from 7 to 56. Responses are scored on a Likert-type scale ranging from 1 (strongly disagree) to 7 (strongly agree), according to how the participant is feeling currently, with a higher score indicating more severe urges [24].

3. Incentive Conflict Task (ICT). ICT is a newly developed task which models contributors to relapse (inherent conflict in abstaining drinkers between the intention to abstain from drinking, and the desire to drink). The ICT requires participants to abstain from responding during presentation of a novel compound stimulus (AB) predicting lack of reward made up of two visual cues that the participant has previously learned to signal reward availability when presented separately (A or B alone). The task requires the participant to respond for the monetary reward, but to withhold responding under conditions in which an increased size of reward might be anticipated (A and B together), but no reward follows. Data are presented as percentage of trials to which participants responded (pressed a key; Table 2). In other words, ICT engages both bottom-up triggers of reward seeking, and the top-down processes that normally modulate and veto responses to such triggers. We have suggested that the task creates a conflict between abstaining and responding for reward similar to that experienced by the participants before relapse, and that the impaired ability of multiple-detoxified participants to perform the task accurately reflects the consequences of detoxification on top-down control of their behaviour [9]. Alcohol-dependent people, as they experience successive detoxifications and their alcohol dependence increases, become increasingly impaired in performing the ICT [25].

4. EQ-5D-5L, an improved version of EQ-5D-3L by Euro-Qol Group [26], is a generic preference-based measure of health- related quality of life that is widely used in health economic evaluations. The descriptive system questionnaire comprises five dimensions: mobility, self-care, usual activities, pain/discomfort and anxiety/depression. Each dimension has 5 levels: no problems, slight problems, moderate problems, severe problems and extreme problems. It was administered with other outcome instruments at baseline, 3, 6 and 12 months. We translated the EQ-5D-5L scores into value sets using Delvin et al.’s approach [27]. Scores from EQ-5D-5L provide a standardised unit of measurement—the quality adjusted life year (QALY)—for use in economic evaluations [28].

5. Self-reported participation in aftercare activities, using a specifically developed log, measuring type and frequency of activity attended, during the period prior the follow-up interview at each follow-up point.

### Process evaluation—treatment fidelity

Observation of 25% of the intervention sessions offered across both recruitment sites using a modified version of the Yale Adherence and Competence Scale version II (YACS II) (2005) was planned [29]. YACS II is a widely used tool that assesses both the frequency/intensity (quantity) and how well CBT techniques (quality) are used in a session, with a score from 1 to 7, with 4 considered as acceptable. Furthermore, 10% of the intervention sessions were planned to be rated by an additional independent rater using YACS II. Additionally, group facilitators were asked to complete a self-assessment form following each session to reflect on their fidelity to the intervention manual. The latter was not completed (see ‘Results’ section).

Audio recording and rating of 25% of the key-working sessions offered to the usual care group was also planned, to assess possible contamination between the study arms, using a specifically developed form based on YACS II items and the main objectives of the SPADe group intervention. The aim was to detect presence of CBT or elements of the intervention within the usual care key working sessions. A low score indicates no presence of specific CBT content and no contamination. The treatment fidelity assessment exercise was particularly important for site 1, where the intervention had already been implemented previously as part of the local treatment pathway.

### Interventions tested

#### SPADe

The intervention provides structured group preparation (additional to usual care) with the aim of helping participants (i) regain partial control over their drinking prior to detoxification and (ii) initiate lifestyle changes for the individual and the immediate family environment. These changes are linked with developing new coping skills and enhanced self-efficacy [30].

The six sessions are numbered and offered weekly in a given order. Stabilisation of alcohol consumption and pattern of drinking is a common theme across all the sessions. To that effect each session can act as an entry point (i.e. an open rolling programme group), despite the special theme covered in depth during the second part of the session. Each session has two facilitators, lasts for one hour and is divided into three parts:

- In the first part (15 min), group rules are established, new members are introduced, as are the aims of the intervention. In-between sessions’ practice allocated in the previous session where individual targets were set are also reviewed.
- The second part (30 min, main part) explores the following themes depending on session number: 1—Understanding habit, addiction and alcohol dependence; 2—Stabilise and control your drinking; 3—Lifestyle changes for you and the people around you; 4—Reduction of your drinking; 5—Achieving abstinence; 6—Relapse prevention strategies. In each session, collaborative activities were completed by the group with facilitators’ support.
- In the third part (15 min), the group summarises the main learning points and agrees in-between sessions’ practice and targets to be achieved before the next session. A group work folder was provided for participants which enabled notes and worksheets to be kept together.

The number of participants per group at any point is between two and eight. This is considered appropriate for theory-based treatment groups, to (i) reach a balance between the educational and treatment elements of the intervention, (ii) promote interactions between group members (group therapeutic effect) and (iii) secure the facilitator’s attention to each member individually [31]. The intervention manual is available online [32]. Participants allocated to the intervention arm entered detoxification at the first available opportunity following completion of the intervention.

#### Usual care

Usual care includes planning for detoxification, detoxification delivery and aftercare conducted in one-to-one keywork appointments. Participants entered detoxification at the first available opportunity (expected to be within 4 weeks from presentation). Whilst waiting for detoxification, they meet their keyworker on 3–4 occasions to maintain motivation and plan aftercare.

Detoxification was medically assisted in the community as an outpatient (both sites), or inpatient (only for site 1), as clinically indicated. The choice depended on health risk factors and availability of social support during detoxification [2]. Participants allocated to Intervention could achieve abstinence by Guided Self-Detox which refers to gradual reduction of drinking guided by the group facilitator during the intervention period. As mentioned above, aftercare (following detoxification) included a relapse prevention group, a small number of individual key worker sessions (4–6 sessions), pharmacological interventions as appropriate peer support groups such as Self-Management and Recovery Training (SMART) Recovery or Alcoholics Anonymous (AA) or more comprehensive aftercare group programme either as an outpatient (both sites) and inpatient (only site 1).

Participants in both intervention and control groups received all the elements of usual care available in the recruiting service. At any time during the trial, change of clinical needs or risks were monitored by the clinical team and participant’s treatment plan was modified accordingly, e.g. urgent hospital admission. These incidents were monitored and reported as per standard ethical recommendations for safety monitoring. Each participant’s care pathway was recorded and analysed for variability within and between sites as part of the economic evaluation (see below), to ensure these were equivalent across trial arms.

### Statistical and health economic analysis

The main analysis was planned to be based on the intention-to-treat principle considering all randomised clients according to the arm they were allocated.

The feasibility outcomes were summarised using descriptive statistics where appropriate. The potential primary and secondary outcomes were summarised by arm, as well as completion rates estimated for each outcome measure. Duration of continuous abstinence, as measured from randomisation, and the time to relapse, as measured from the end of the detoxification. Formal hypothesis tests and confidence intervals have not been reported due to the nature of this trial as the focus was on the completion rate of outcomes, and the estimation of parameters required for a sample size calculation for the main trial.

Economic evaluation in the feasibility study tested the collection of data on costs and outcomes that would be required in any future definitive trial assessing the cost effectiveness of the SPADe intervention. Data gathered on the resources-use involved the delivery of the intervention and on service use by participants over the 12-month period of the study. Clinic contacts with key workers, nurses, and doctors for delivering the intervention and usual care were obtained retrospectively at 3, 6 and 12 months from clinic records by research assistants. The records covered all participants’ contacts including face-to-face and by phone with health professionals. For those in the intervention group, the clinic records included attendances at the detoxification preparation sessions. These contacts were subsequently separated out as part of the analysis, in order to assess compliance and isolate other service use. To assess potential service use offsets, data on other service utilisation by participants was gathered by self-report using the Client Service Receipt Inventory. The CSRI was administered to participants by researchers in the clinic at 3, 6 and 12 months. The questionnaire covers all forms of health and social care and includes contacts with the police and justice services [33]. The data on contacts with health and social care professionals and service-use were converted to costs by multiplying contacts by nationally validated unit costs (which include on costs and facilities and managerial overheads) in British pounds 2018 [34]. These data from clinic records included the duration of contacts (in minutes) which were used for the cost calculation. Unit costs for legal services and police contacts were obtained from the Home Office and related sources [35].

### Qualitative interviews

A purposive sample of participants across both trial groups and sites (approximately 20) were planned to be interviewed at 3 months to establish experiences of randomisation, recruitment and initial trial procedures (wave 1) and follow up interviews were planned to take place at 9 months (completion of the study, wave 2) to give specific feedback on retention issues and treatment conditions. Due to difficulties interviewing participants at two time points, interviews were conducted at any time between 6 and 9 months from recruitment into the study. A selected sample of staff involved with the intervention (both directly and in-directly) were also interviewed on study completion.

### Sample size

No formal calculation is required for a feasibility study since measuring effectiveness was not a key objective. The sample size of 50 was considered appropriate for assessing key objectives related to recruitment, retention, randomisation, data capture, performance of outcome measures and acceptability. In particular, this would allow us to estimate the retention of the participants to within ± 11% assuming an 80% retention rate and is in line with current guidelines to estimate the parameters required for a sample size calculation [36].

### Randomisation and masking

Following written informed consent, participants were randomised using a third-party web-based randomisation system which ensured concealed allocation. Participants were stratified according to number of previous detoxifications (> 2 vs. ≤ 2) and site. Randomisation had a random block size (2–4). Research Assistants were blind to the randomisation occurring at the opposite study site. It was planned for them therefore to conduct the follow-up interviews for the opposite site. This initial plan for cross site follow-up was abandoned during the 3 months follow-up period as it proved to be too great a logistic and administrative barrier for successful follow up efforts.

#### Progression criteria

Progression criteria to a full RCT were agreed in advance and included study retention, treatment compliance and data completion.

## Results

### Demographics

We recruited alcohol-dependent individuals, aged 18 or over who had a desire to stop drinking. Table 1 shows the baseline data for all 48 randomised participants. Overall, whilst the two groups were reasonably equivalent in terms of mean age, ethnicity, years of education and key variables such as baseline SADQ, there were also important differences in sex and living arrangements with more males and single people in the intervention group. However, this is not unexpected given the size of the groups. Overall, the participants had an average age of 46.4 years (range, 31 to 63), 28 (58%) were male and had previously undertaken a median of two previous detoxifications (range, 0 to 24). These demographics reflect the population in treatment.

**Table 1:** Baseline characteristics of randomised participants

Age (years), mean (SD)	46.70 (9.82)	46.16 (10.94)
Gender, *n* (%)		
Female	13 (57%)	7 (28%)
Male	10 (43%)	18 (72%)
Ethnicity, *n* (%)		
African	1 (4%)	0 (0%)
Any other Black background	1 (4%)	1 (4%)
Any other ethnic group	0 (0%)	1 (4%)
Any other white background	3 (13%)	1 (4%)
British	12 (52%)	14 (56%)
Caribbean	0 (0%)	1 (4%)
Indian	5 (22%)	3 (12%)
Irish	1 (4%)	3 (12%)
Pakistani	0 (0%)	1 (4%)
Migrant, *n* (%)	11 (48%)	9 (36%)
Years of education, mean (SD)	11.57 (2.09)	11.88 (2.39)
Highest qualification, *n* (%)		
A Level/NVQ	2 (9%)	7 (28%)
Diploma/BTEC	2 (9%)	0 (0%)
No qualification	4 (17%)	3 (12%)
O level / GCSE	11 (48%)	9 (36%)
Other (please specify)	1 (4%)	0 (0%)
University Degree	3 (13%)	6 (24%)
Employment status, *n* (%)		
Full time	4 (17%)	3 (12%)
Part time	2 (9%)	3 (12%)
Sick leave > 4 weeks	4 (17%)	2 (8%)
Unemployed	13 (57%)	17 (68%)

### Feasibility outcomes

#### Recruitment

**Fig. 1:**
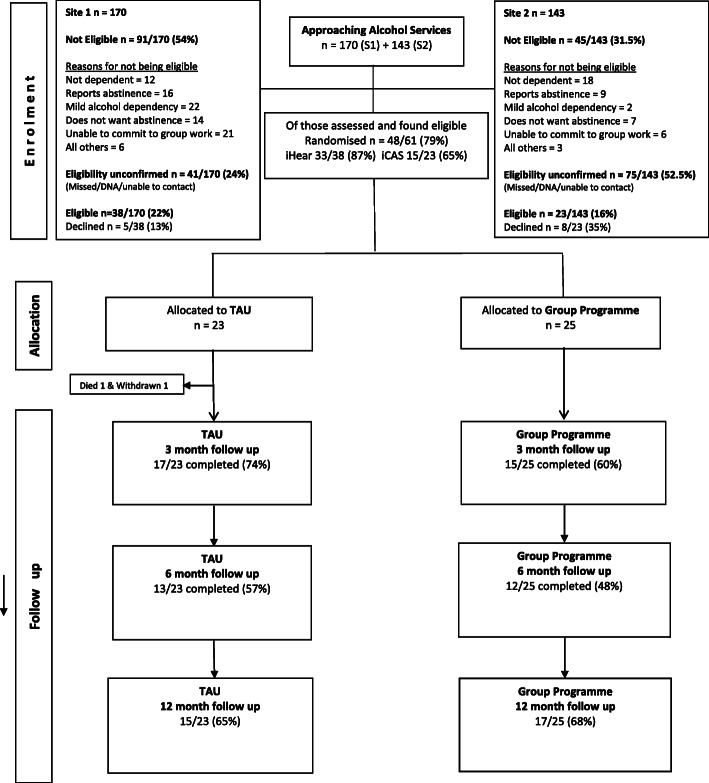
Trial consort diagram by treatment arm

Forty-eight out of fifty eligible participants were recruited over 9 months (September 2017–May 2018). Thirty-three participants were recruited from site 1 and 15 from site 2. The main reasons for not been eligible to participate were not wishing to be abstinent and inability to commit to group work (see Fig. 1). The rate of recruitment was higher when the research assistants were on site, which was supported by the qualitative interviews of staff (see section below).

#### Retention

Retention in the trial for the whole duration of the 12 months was 75%, 80% (*n* = 20) for the intervention group and 69% (*n* = 16) for usual care. The retention rate indicates strong acceptability in the study population (see Table 2).

**Table 2:** Feasibility outcomes and secondary (incentive conflict task; ICT) outcomes; 1 due to technical problems only three participants completed ICT after baseline at 3 months and three at 12 months, thus only baseline measurements are presented for ICT

Outcome	Control (*n* = 23)	Intervention (*n* = 25)	Overall (*n* = 48)
Recruitment rate, number per month (95% CI)			6 (4.42,7.96)
Trial retention rate	16 (69.6%)	20 (80.0%)	36 (75.0%)
Compliance			
Site 1		8/17 (47.0%)	
Site 2		3/8 (37.5%)	
Completeness			
Days of abstinence			
3 months	17 (74%)	14 (56%)	31 (65%)
6 months	12 (52%)	12 (48%)	24 (50%)
12 months	14 (61%)	16 (64%)	30 (63%)
SADQ			
3 months	15 (65%)	14 (56%)	29 (60%)
6 months	7 (30%)	8 (32%)	15 (31%)
12 months	12 (53%)	13 (52%)	25 (52%)
Alcohol urge			
3 months	15 (65%)	13 (52%)	28 (58%)
6 months	7 (30%)	7 (28%)	14 (29%)
12 months	11 (48%)	11 (44%)	22 (46%)
Secondary measurements			
ICT measurements at baseline^1^: Number of presses (%) to obtain reward [mean (SD)]			
			Overall (n=20)
In the presence of single stimuli A or B predicting reward	-------	-------	82.8 (32.8)
In the presence of combined stimuli AB predicting lack of reward	-------	-------	63.3 (37.9)

#### Follow-up

The follow-up rate was 65% (*n* = 31) at 3 months, 50% (*n* = 24) at 6 months and 63% (*n* = 30) at 12 months, with more follow-ups taking place over the phone. The follow-up rate at 3 months was 74% (*n* = 17) for usual care and 56% (*n* = 14) for the intervention. At 6 months, it was 52% (*n* = 12) for usual care and 48% (*n* = 12) for the intervention. Finally, at 12 months, it was 61% (*n* = 14) for usual care and 64% (*n* = 16) for the intervention (Fig. 1).

#### Intervention sessions attended

Treatment compliance, defined as attending 6 sessions over 12 weeks, was 44% with 47% (8/17) and 37.5% (3/8) for the first and second site respectively, which is considered acceptable for a population attending addiction services [13].

#### Progression criteria

Progression criteria to a full RCT were agreed in advance and included study retention, treatment compliance and data completion. As shown in Table 3, retention was excellent, and no change is required. Regarding treatment compliance, minor changes are required to proceed to a full trial, whereas regarding data completion for primary outcome major changes are required before proceeding to a full trial.

**Table 3:** Study progression criteria

	Proceed	Proceed with changes	Do not proceed without major changes
Compliance with the intervention	≥ 60%	40–59%	< 40%
Retention of participants in trial	≥ 75%	50–74%	< 50%
Completion of primary outcome data in participants not lost-to-follow-up	≥ 90%	80–89%	< 80%

#### Data completeness

Completeness of data outcomes was varied (29% as lowest for Alcohol Urge Questionnaire at 6 months to 74% for days of abstinence at 3 months). Completeness of primary outcome was 65%, 50% and 63% at 3-, 6- and 12-month follow-up respectively.

Completion of the Incentive Conflict Task (ICT) was compromised with only 13 tests completed at three 3 months and even less at all three follow-up points. This was direct result of the need for most participants to be followed-up over telephone and not face to face as initially planned (see Table 2).

#### Primary outcomes

The intervention group had more days abstinent in the previous 90 days at 12 months (54.5) versus usual care (41.5). Seven intervention participants (28%) and five of the usual care group (22%) restarted drinking at some point during the follow up period.

#### Secondary outcomes

Severity of dependence (SADQ scores) were higher, time to relapse shorter and urges (Alcohol Urge Questionnaire scores) higher for the intervention group at all follow up points. The results from ICT will be reported separately. A summary is presented in Table 2.

Equal percentages (52%) from each arm were detoxed (including guided self-detox). This is lower than anticipated, possibly due to generic challenges that services face.

#### Health economics evaluation

Of the total of 48 participants, 39 were included in the health economic analysis (19 intervention and 20 usual care). Of the nine excluded, one had moved from intervention to usual care outside of study protocol procedures and the clinic records for eight in one site were missing due to a facility relocation. Clinic data were not complete for many of the remaining 39 participants with more items missing in the usual care than intervention group. Approximately 50% of participants in both groups provided 12 months follow-up questionnaire data (service use and EQ-5D). These levels of missing data were considered too high to provide accurate estimates of costs or changes in health-related quality of life. Two key workers delivered each session of the intervention at a cost (2018) of £102 per session (£17 per participant per session), based on six per group, and NHS unit costs [34].

The mean time spent with participants by keyworkers was 230.5 min in the usual care group and 340.84 min for the intervention group. Converting service use into costs, based on mean durations of contact, gives mean total costs of £356.81 for the intervention group, compared to £254.69 for usual care group. The intervention group reported more community service use than the usual care group. The overall costs are reflective of contacts with healthcare professionals: £947.54 for the usual care group compared to £1933.62 for the intervention group, which includes costs for attendance at preparatory sessions.

#### Process evaluation-treatment fidelity

At least one of each of six group sessions were observed and scored independently for compliance with the manual, by the Chief Investigator (CK) and an independent rater (nurse Consultant in Addiction services), using YACS II (2005). Group facilitators did not rate their own fidelity as per the original protocol. Instead, the group facilitators were interviewed individually by CK following the end of the intervention provision period for the study. As far as usual care was concerned, tape recording of key working sessions offered as part of usual care was done in site 1 only. Nine of those sessions were assessed in total, with at least one of each of the three planned sessions and at least one session per keyworker, using a specifically developed tool (see above). In the second site, keyworkers refused to be taped. To that effect, medical notes were reviewed by CK and the content of session was scored using the same assessment tool. Scores suggested no contamination of key working session with content from the intervention in site 1. In site 2, 3/7 participants allocated to usual care, received additional sessions of psychological work beyond Motivational Interviewing, either by their keyworker or a psychologist.

#### Qualitative interviews

Fourteen participants were interviewed from both sites. Five of those were interviewed twice (between 3, 6 and 12 months) as per the original protocol, providing in total 19 interviews. Interview guides were constructed taking a narrative approach, asking participants to ‘tell the story’ of their history of alcohol use, previous treatment episodes, detoxification attempts, events that led up to the current treatment episode, experiences of taking part in the intervention/control preparation groups, experiences of the actual detoxification, then subsequent recovery, adaptation or relapse experiences. Interviews were conducted by experienced researchers trained in qualitative interviewing techniques (BH, CN). Interviews lasted 60–90 min. Audio files of interviews were transcribed verbatim and anonymised. Data were inductively thematically analysed case by case independently by both BH and CN using QSR NVIVo v12 software. Descriptive thematic analysis was the most appropriate analysis technique for answering feasibility questions [37].

Overall, the qualitative data was generally positive and supportive of the study. With a few exceptions, participants understood the purpose of the study and were willing to be randomised. It was noteworthy that there was some confusion regarding group allocation. This suggests that simply ‘being in the study’ had a positive benefit. Also, of note was the positive feedback for the preparation groups (intervention). However, the interviews gleaned little detailed data on CBT-specific elements of the group intervention. Instead, participants had generally positive feedback that seemed to be related to a generalised group effect (being part of a group, sharing experiences and learning from others). Most individuals found this beneficial, with only a few exceptions (notably those with diagnosed mental health conditions who found group participation difficult).

Two staff members were interviewed (one from each site) following the end of the study. Staff were generally supportive of the intervention; however, staff acceptability of both content and structure was key in consistent facilitation of the intervention. Staff were initially hesitant to use novel pre-habilitation strategies until they had seen results for themselves and then understood how it was useful to participants. There were some practical issues raised—availability of rooms and room set up as being important to the running of the groups. There was a lot of discussion about re-tendering of treatment services and collection of outcome measures which may conflict with ‘contract-led’ key performance indicators for joint service providers. These discussions initially impeded smooth service running and group facilitation until it was evident that the intervention facilitated achievement of key performance indicators for the service providers. This is an important context for understanding difficulties with implementing the group intervention.

## Discussion

This study reports the feasibility of conducting a trial to further enhance the psychological work offered before alcohol detoxification within the overall concept of pre-habilitation, to complement the existing rehabilitation treatment approach.

We have recruited from two urban clinical addiction sites, with easy access to public transport. Both sites were covering a multicultural population, where language could be a barrier for accessing treatment, even more so when accessing a group intervention. In this feasibility study, we did not need to offer the intervention on an individual basis due to language barriers (as per protocol); nevertheless, language remains a barrier for a group intervention. Limited transport and longer duration to and from the place that the group is offered could be a barrier, as it could prolong the period of non-drinking required and associated risk of withdrawal symptoms. Another important factor related to the generalisability of the intervention concerns differences in service models and commissioning arrangements between the recruitment sites. Such differences might be challenging for the standardisation of the usual care arm of the study; nevertheless, the challenges faced enhance the wider generalisability of the results. In this feasibility study, there was a major difference between the two sites on the procedures for access to inpatient detoxification. In site 1, the decision and the budget were controlled by the local team, whereas in site 2 the decision and budget allocated involved commissioners and specific steps had to be followed (which did not allow randomisation). This meant that only participants requesting outpatient detoxification were entered into the study in site 2. Remaining procedures across sites were standardised.

We were able to recruit appropriate participants (moderate to severe dependence on alcohol) with a rate of six recruits per month. The recruitment rate has been compromised by the generic environment of the addiction services in England such as limited investment, frequent re-tendering of services and an overall climate of competition rather than collaboration [37]. Such factors could become barriers for successful recruitment for a future RCT trial. Nevertheless, the sample demographic characteristics reflected those of the population in treatment [13]. Randomisation was successful except for more males and single people having been allocated to the intervention arm; however, neither were considered to be important confounders likely to affect the results.

The retention rate was particularly good for dependent alcohol users. Similarly, follow-up rates at 12 months for the primary outcome were also acceptable. Low follow-up rates were observed at 6 months which coincided with the immediate period prior and post re-tendering in the highest recruiting site (site 1). Interview over telephone was found to be the best follow up strategy but compromised the ability to collect data for ICT, CSRI and other questionnaires, indicating the importance of using brief tools rather than longer self-completion questionnaires. A mixed method of CSRI and clinical notes review should be used for health economic evaluation. There were also technical difficulties with the operation of ICT. Despite the above challenges, this has been the first time that ICT is used with persons whilst they are still drinking (23 participants at baseline), and this study provides valuable data on the effect of alcohol in decision making. These results will be reported separately. Data completion though for the primary outcome was lower than expected suggesting major changes are required. Equally, we consider a longer recruitment period with full time research assistants positioned in each recruiting site would be more effective than seeking larger numbers of recruiting sites with either part time or full-time research assistants recruiting for shorter recruitment periods. Such a strategy might compromise the generalisability of the results. This risk could be reduced with better triangulation of recruiting sites taking into account two main factors (i) geography (urban/rural), and (ii) commissioning arrangements.

To allow for better monitoring of study participants, research assistants were blind to randomisation occurring at the opposite site. The original plan was for research assistants to complete follow-ups at the opposite site and hence to be blind to allocation. However, blinding for psychological interventions is difficult with this population [2]. Blinding proved to be over ambitious for a trial with this population where appointments were missed and subsequent follow up was further complicated by research assistant availability.

Compliance with the intervention although acceptable has indicated that changes are required for a definitive trial. We consider that recruitment and randomisation at the first point of contact with the service increased the risk of early drop out from treatment and the study, compromising treatment compliance. Furthermore, in the current study, two individuals with long histories of poor treatment retention and early dropouts were allocated to the intervention arm. In a future RCT, we would randomise individuals at a later point once they are retained in the service for a brief period (usual practice of 4 weeks), having received a small number of key working sessions and so demonstrating some clear commitment to treatment. Furthermore, the intervention should be better standardised with the expectation that participants attend consecutive weekly sessions, within a maximum period of 12 weeks. Missed sessions would be offered by one of the facilitators on an individual basis. Most importantly there would be more enhanced communication and cooperation between key workers and group facilitators for a pro-active and more effective management of participants at risk of dropping out.

The strategy used to assess fidelity to the intervention manual was adequate as was the training, supervision and support provided to group facilitators for the study. For a future trial, support of group facilitators should be allocated to local supervisors per recruitment site to increase hands-on support. This is considered critical to reduce contamination between the intervention and usual care.

The low rate of participants receiving detoxification for both arms could be an indicator of limited progression of clients through treatment stages, highlighting the major challenges that services face in their efforts to deliver treatment in a timely and structured way. Generic factors in English addiction services such as tendering of services, limited resources and high turnover of staff are compromising the capacity of treatment services to host clinical research and are also compromising the standardised delivery of both intervention and usual care. These factors have been discussed widely both in the past and more recently in the UK [38]. In a future trial, the ongoing support to group facilitators and the monitoring of usual care provision should be augmented, given the challenging clinical reality.

## Conclusions

Psychological interventions with individuals who are dependent on alcohol and who are actively drinking at the time of the intervention have been regarded as challenging. Furthermore, limited work has been done so far on their effectiveness and cost-effectiveness. The results of this feasibility trial indicate that despite the challenges described above, with appropriate modifications, a full multicentre trial would be possible to test the effectiveness and cost-effectiveness of a pre-habilitation-based intervention such as SPADe group against usual care.

## Data Availability

The datasets generated and/or analysed during the current study are available from the corresponding author on reasonable request.
